# A Comparative Study of Dorsal Buccal Mucosa Graft Substitution Urethroplasty by Dorsal Urethrotomy Approach versus Ventral Sagittal Urethrotomy Approach

**DOI:** 10.1155/2013/124836

**Published:** 2013-09-30

**Authors:** Mrinal Pahwa, Sanjeev Gupta, Mayank Pahwa, Brig D. K. Jain, Manu Gupta

**Affiliations:** ^1^Sir Ganga Ram Hospital, New Delhi, India; ^2^Army Research and Referral Hospital, New Delhi, India

## Abstract

*Objectives*. To compare the outcome of dorsal buccal mucosal graft (BMG) substitution urethroplasty by dorsal urethrotomy approach with ventral urethrotomy approach in management of stricture urethra. *Methods and Materials*. A total of 40 patients who underwent dorsal BMG substitution urethroplasty were randomized into two groups. 20 patients underwent dorsal onlay BMG urethroplasty as described by Barbagli, and the other 20 patients underwent dorsal BMG urethroplasty by ventral urethrotomy as described by Asopa. Operative time, success rate, satisfaction rate, and complications were compared between the two groups. Mean follow-up was 12 months (6–24 months). *Results*. Ventral urethrotomy group had considerably lesser operative time although the difference was not statistically significant. Patients in dorsal group had mean maximum flow rate of 19.6 mL/min and mean residual urine of 27 mL, whereas ventral group had a mean maximum flow rate of 18.8 and residual urine of 32 mL. Eighteen out of twenty patients voided well in each group, and postoperative imaging study in these patients showed a good lumen with no evidence of leak or extravasation. *Conclusion*. Though ventral sagittal urethrotomy preserves the blood supply of urethra and intraoperative time was less than dorsal urethrotomy technique, there was no statistically significant difference in final outcome using either technique.

## 1. Introduction

Strictures of anterior urethra are commonly idiopathic or occur following balanitis xerotica obliterans, faulty catheterization, instrumentation of urethra, and pelvic injury. Short strictures (<3 cm) have been managed by end-to-end anastomosis of urethra with almost 100% success rate. However, reconstruction of stricture greater than 3 cm often leads to chordee and impotence as the length of the stricture increases [1]. Hence, long strictures have been treated by graft substitution urethroplasty [2]. Various genital and extragenital grafts have been used for substitution urethroplasty [3]. But they carry the disadvantage of higher chances of graft necrosis leading to recurrence and donor site morbidity [4]. Buccal mucosa graft (BMG) has emerged as a versatile substitute because of easy harvest, resilience due to thick epithelium and rich elastin content, and good take [5], though it is associated with complications of pain, numbness, and restriction of mouth opening [6–10]. Graft bed heals rapidly with minimum postoperative morbidity. In addition, BMG is resistant to infection and trauma [5]. Initially ventral substitution urethroplasty came in vogue because of simplicity of access and excellent graft bed offered by spongy tissue. Stricture was easily visualized and lumen was clearly delineated [8]. But it fell into disrepute because of ballooning, sacculation, and urethrocele leading to postvoid dribbling. Urine stasis in ballooned graft led to urinary infection and fistula formation [11–13]. Other complications are shrinkage of graft because of lack of mechanical support or insufficient graft neovascularization. Dorsal substitution urethroplasty is devoid of the above said complications. It can be performed by two approaches: dorsal urethrotomy [14–16] and ventral sagittal urethrotomy [17–20]. In this series we have attempted to assess and compare the following.

1. Feasibility and efficacy of both of the approaches.
2. Postoperative results.
3. Complications.

## 2. Materials and Methods

From June 2005 to May 2011, 40 patients with stricture of anterior urethra underwent one stage BMG substitution urethroplasty. Inclusion criterion included any penile, penobulbar, and bulbar stricture of any etiology except traumatic. Patients with pan urethral stricture greater than 12 cm and those associated with infection, high bulbar strictures, completely obliterated stricture with insufficient urethral plate, and previously failed urethroplasty were excluded. The Ethics Committee of the hospital approved this study, and the patients signed their consent on a written form of information. All patients were investigated with urine culture and sensitivity, retrograde urethrogram and micturating cystourethrogram (RGU-MCU), ultrasound kidney, ureter, and bladder (KUB), and postvoid residual urine and uroflowmetry. Patients were divided in two groups: Group A underwent dorsal BMG substitution urethroplasty by dorsal urethrotomy approach and Group B by ventral sagittal urethrotomy. Patients were randomized using computer generated randomized tables. 

Patients were advised to stop smoking and chewing tobacco six weeks prior to surgery and were put on regular mouth washes with chlorhexidine 2 days prior to surgery and continued for another five days postoperatively. Patients were administered broad spectrum antibiotics (ceftriaxone and amikacin) before starting surgery which were continued for another three days followed by oral antibiotics for another week. Transnasal intubation was carried out to facilitate harvesting of BMG. Urethroplasty was performed by two teams: one harvesting and preparing the graft and the other exposing the stricture and finally doing the anastomosis. The length of the defect was measured and the graft accordingly harvested from inner cheek. The donor site was left unsutured, and local anesthetic was administered along with adrenaline gauze compression to control the bleeding. 

**Figure 1:**
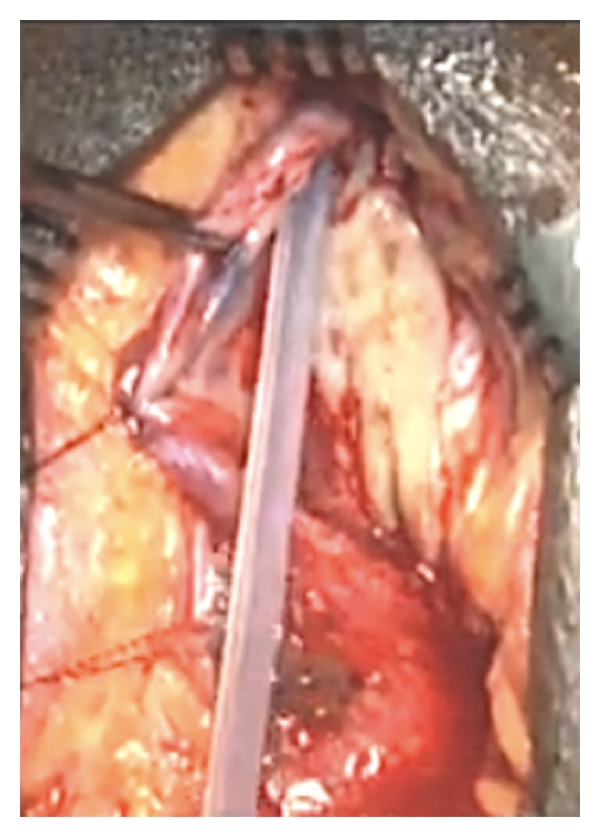
BMG placement in Barbagli technique.

All patients underwent uretheroscopy before commencing surgery. Penile urethra was exposed by circumcoronal incision and degloving of skin. Bulbar urethra was exposed by midline perineal incision or inverted Y-shaped perineal incision for the strictures of bulbar urethra. In Group A, urethra was exposed, mobilized, and rotated to 180 degree. After dorsal urethrotomy of strictured urethra, BMG was sutured to the bed of corpora cavernosa site with 4/0 vicryl over 16 Fr silicone catheter (Figure 1). Quilting was done to prevent shrinkage and displacement of graft. Later, free margins of preplaced buccal graft were sutured to the respective edges of dorsal urethrotomy. 

**Figure 2:**
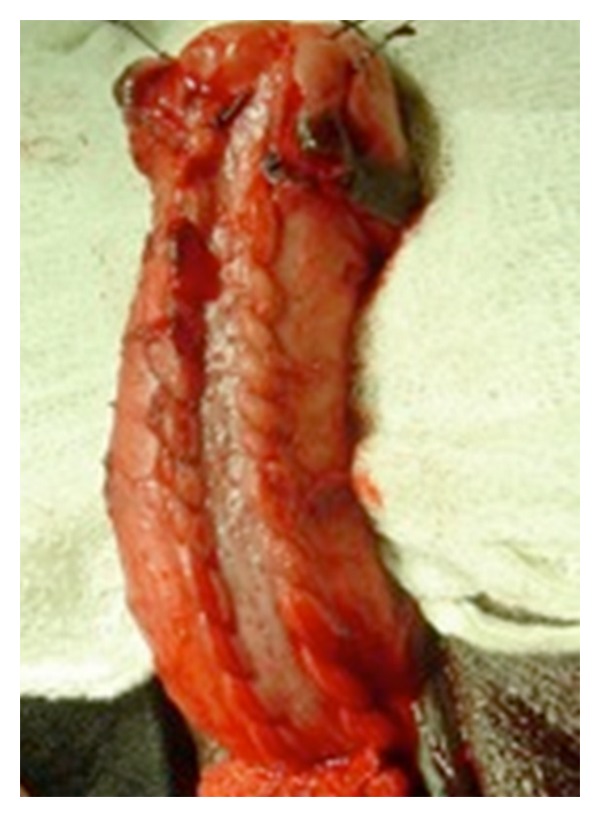
BMG placement in Asopa technique.

In Group B, after exposing the urethra, ventral urethrotomy was done at stricture site. Stay sutures were taken, and dorsal urethrotomy was done. Cut edges of dorsal wall were separated from tunica albuginea by blunt dissection along the entire length of stricture with handle of scalpel to make elliptical raw area. Buccal mucosal graft was sutured to the free edges with 4/0 vicryl (Figure 2). Intervening sutures were placed between graft and corporal bodies in quilted manner to reduce dead space. Ventral urethrotomy was closed with 4/0 vicryl over 16 Fr silicone catheter, which worked as a splint for the graft. Suprapubic cystostomy (SPC) was placed in all patients irrespective of the approach.

Patients were advised to bed-rest for a week after surgery and were allowed to do light work thereafter. Urethral catheter was removed on 10–14 postoperative day. SPC was removed in next few days after confirming satisfactory voiding. All patients were followed at 3, 6, 9, and 12 months postoperatively. At every visit, patients were assessed for symptoms and comfort level and underwent urine culture and sensitivity, ultrasound KUB, and post void residual urine and uroflowmetry. All patients who complained of poor flow and with suboptimal uroflowmetry (*Q*_max⁡_ < 15 mL/msec) underwent RGU-MCU. Urethroplasty was considered a failure if any operative intervention was performed in the postoperative period.

## 3. Results

The age of the patients ranged from 17 to 80 years with mean age of 40 years. The preoperative characteristics of the study population have been defined in Table 1. Most common presentation was poor urinary flow with concomitant straining at micturition. 10 patients presented with acute urinary retention for which SPC was done. The most common site of stricture was bulbar urethra (22 cases) followed by penobulbar (10 cases) and penile urethral stricture (8 cases). Length of stricture varied from 3 to 12 cm with mean of 6.8 cm. The etiology of the stricture was infection (including lichen sclerosus) in 42.5%, iatrogenic in 27.5%, idiopathic in 20%, and trauma in 12.5%. Although not statistically significant, the intraoperative time was found to be lesser in Group B as compared to Group A (142 versus 125 min, *P* = 0.67). The patients were followed for 6–24 months with mean follow-up of 12 months.

Table 2 shows the postoperative results of both of the groups. In Group A, 18 patients were satisfied with surgery and had *Q*_max⁡_ greater than 25 mL/sec. One patient had persistent narrowing at the stricture site and voided with a poor stream. Patient was managed with optical internal urethrotomy (OIU). Second patient had accidental removal of urethral catheter on second postoperative day and mild perineal wound gaping. SPC was removed in this case after two weeks. Patient was voiding satisfactorily in immediate postoperative period. Patient developed poor urinary stream at 4 months of follow-up. RGU/MCU revealed significant narrowing at operated site. In uroflowmetry, *Q*_max⁡_ was 12 mL/sec with PVR of 200 mL. He was managed with OIU and was voiding satisfactorily in further follow-up. In Group B, all patients did well in the postoperative period after catheter removal except one who had developed postoperative wound infection which was managed with injectable antibiotics. On subsequent follow-up, 2 patients showed evidence of stricture at 1 year in Group B. Both of these patients were managed with OIU. There was no significant difference in the subjective symptom score, residual urine, *Q*_max⁡_, and restricture rate between the two groups.

The complications observed in both of the groups were as shown in Table 3. Most of the complications observed were clavien grade I/II in both of the groups. Only two recurrent strictures required operative intervention in the form of OIU in Group A, and one fistula repair and two OIU were done in Group B making them grade III complications. In total, 12 complications were observed in 7 patients in group A and in 8 patients in group B.

## 4. Discussion

Long stricture urethra (>3 cm) requires graft interposition to prevent chordee and impotence [1]. Buccal mucosal graft has emerged best amongst other possible grafts available from various sites because of good take, resilience, and easy harvest [5]. Grafts can be placed dorsally [14–16] or ventrally [17–20] at strictured site of urethra. Multiple studies have shown that both ventral and dorsal onlay BMG have good blood supply and mechanical support. Barbagli showed that the success rate is equal with dorsal and ventral BMG [18]. The technical advantages of ventral onlay are considerable. Strictures are easily visualized. The lumen is clearly delineated with urethrotomy, allowing the surgeon to identify mucosal edges, measure the size of the plate, carry out a water-tight anastomosis, and, if necessary, excise portions of the stricture and perform dorsal reanastomosis. Ventral onlay has been criticized because of excessive blood loss and a high incidence of diverticulum formation. With a healthy spongiosum, bleeding is expected. Limitations to ventral onlay urethroplasty include severe spongiofibrosis due to prior failed urethroplasty or pelvic irradiation and strictures of the distal penile urethra. Spongiosum is not abundant, and spongioplasty is difficult to achieve. 

The dorsal approach to treating strictures of the bulbar urethra is anatomically sounder than the ventral approach because it requires less extensive opening of the spongy tissue; the urethral lumen is located dorsally [2]. The dorsal approach avoids significant bleeding from the corpus spongiosum, and mechanical weakening of the graft is unlikely. A serious complication of free graft urethroplasties is necrosis of the patch, which is caused by the failure of vascularization from its bed. When this occurs in ventrally placed grafts, an urethra-perineal fistula of considerable size is inevitable. No such event occurs in patients treated using a dorsal graft apposition [2]. The dorsal placement of the graft provides a potential for roof-strip epithelial regeneration, according to the standard laid down principles of urethroplasty [21–23], provided that a catheter is left indwelling for an adequate period of time. 

Dorsal urethroplasty can be performed by two techniques [11–13]. Dorsal urethrotomy requires exposure, dissection from tunica albuginea, and rotation of strictured urethra [14–16]. This technique takes longer operative time, and it causes more blood loss though not amounting to significant fall in post-op haemoglobin and possibility of ischemia due to mobilization of urethra involving injury to circumflex and perforating vessels. Novel techniques have been described to circumvent this problem. Kulkarni et al. investigated the feasibility of a new one-sided anterior dorsal onlay oral mucosa graft urethroplasty while preserving the lateral vascular supply to the urethra, central tendon of the perineum, the bulbospongiosum muscle, and its perineal innervation. Of the 24 patients, 92% had a successful outcome [24]. The same technique was also found to be effective by Chaudhary et al. who carried out urethroplasty by the perineal incision, accessing pendulous urethra by penile eversion through the same incision. They found decreased incidence of chordee, wound infection and fistula formation [25]. We observed similar success rates of 90% while using this technique. While analyzing outcomes and complications in a large cohort of 163 patients, Hoy observed that 96.9% had no evidence of stricture. Postoperative complications included postvoid dribbling (41.7%; 68 of 163), urinary tract infection (3.7%; 6 of 163), erectile dysfunction (3.1%; 5 of 163), orchalgia (10.4%; 17 of 163), and donor site morbidity (4.3%; 7 of 163). The complication rate in our series was 35% for all the complications.

Asopa et al. explored a ventral sagittal approach for dorsal onlay BMG urethroplasty techniques [17]. The urethra was not separated from corporal bodies and was opened in the midline over the stricture. The floor was incised, and an elliptical raw area was created over the tunica on which a full thickness graft of preputial or buccal mucosa graft was secured. After a follow-up of 8–40 months, only one patient out of 12 developed recurrence. The same technique has been well studied, and equivalent success rates have been shown [26, 27]. We observed a success rate of almost 90% using Asopa technique with satisfaction rate of 95% at three months and 90% at one year. Four out of twelve patients developed complications in the cohort of patients studied by Asopa, namely, hematoma, fistula, recurrent stricture, and chordee formation. In another series using Asopa technique, the authors reported 21 complications in a case series of 58 patients. Seven patients had wound infection, five had urethrocutaneous fistula, six had recurrence, and three had donor site complications. We had similar complication rate although the complication profile differed in our case series. The infection rate was lesser in our series due to heavy and broad-spectrum antibiotic coverage.

In the present study, Asopa technique was found to take lesser time to perform as compared to the Barbagli although the difference was not statistically significant (142 versus 125 minutes, *P* = 0.69). Asopa technique was also found to be more suitable in cases where anterior urethra was found to be densely adhered to corpus cavernosum due to extensive spongiofibrosis. The complication rate was found to be equal in both of the approaches. In this series, 18 out of 20 patients were voiding satisfactorily at one-year follow-up in each group. Patients who failed in both of the groups were easily managed with OIU. The main limitation of our study were the short follow-up, smaller number of patients, and heterogeneity of our study population in terms of site of stricture and etiology of stricture. 

## 5. Conclusion

Dorsal onlay buccal mucosal graft substitution urethroplasty for all morbid urethral strictures is feasible by both approaches. However, for purely penile urethral strictures ventral urethrotomy approach may be preferred due to easy accessibility to urethra and less time consuming, although larger randomized studies with longer follow-up are necessary before making a definite recommendation.

## Figures and Tables

**Table 1:** Preoperative evaluation.

	Age (in years)	Length of stricture (cm)	RGU/MCU-site of stricture	Uroflowmetry *Q*_max⁡_ (mL/sec)	USG KUB/PVR (mL)
Group A	R: 17–69 M: 38.5	R: 3–12 M: 6.5	Bulbar: 12 Penobulbar: 4 Penile: 4	R: 1.5–10 M: 4.5	R: 100–300 M: 180
Group B	R: 22–80 M: 41.2	R: 3.4–11 M: 7.2	Bulbar: 10 Penobulbar: 6 Penile: 4	R: 3–13 M: 5.8	R: 120–360 M: 230

R: range, M: mean.

**Table 2:** Postoperative evaluation.

	Group A		Group B	
	Postoperative	At 1 year	Postoperative	At 1 year
Subjective assessment of symptoms				
Excellent	18/20	18/20	19/20	18/20
Average	2/20	2/20	1/20	2/20
*Q*_max⁡_	26.2	19.6	25.8	18.8
RGU/MCU				
Normal	18/20	18/20	19/20	18/20
Stricture	2/20	2/20	1/20	2/20
Residual urine	13.5 mL	27 mL	22 mL	32 mL

**Table 3:** Complications as observed groupwise.

Complications	Group A	Group B
UTI	1 (5%)	2 (10%)
Postvoid dribbling	4 (20%)	4 (20%)
Urethro cutaneous fistula	0	1 (5%)
Erectile dysfunction	3 (15%)	2 (10%)
Donor site complications	1 (5%)	1 (5%)
Recurrence	2 (10%)	1 (5%)
Wound infection	1 (5%)	1 (5%)
